# Gaze and body cues interplay during interactive requests

**DOI:** 10.1371/journal.pone.0223591

**Published:** 2019-10-21

**Authors:** Sonia Betti, Umberto Castiello, Silvia Guerra, Umberto Granziol, Giovanni Zani, Luisa Sartori

**Affiliations:** 1 Department of General Psychology, University of Padua, Padua, Italy; 2 Padova Neuroscience Center, University of Padua, Padua, Italy; Birkbeck University of London, UNITED KINGDOM

## Abstract

Although observing other’s gaze and body movements provides a crucial source of information to successfully interact with other people, it remains unclear whether observers weigh differently these cues and whether the convergence of gaze and body’s directions determines facilitation effects. Here we aim to shed more light on this issue by testing the reliance upon these cues from both a behavioral and a neurophysiological perspective in a social interactive context. In Experiment 1, we manipulated the convergence between the direction of an actor’s upper limb movement and gaze direction while he attempts to socially interact with the participants observing the scene. We determined the direction of gaze as well as the duration of participants’ ocular fixations during the observation of the scene. In Experiment 2, we measured and correlated the effect of the body/gaze manipulation on corticospinal excitability and on the readiness to interact—a disposition to engage in social situations. Eye-tracking data revealed that participants fixated chiefly the actor’s head when his hand and gaze directions were divergent. Possibly a strategy to disambiguate the scene. Whereas participants mainly fixated the actor’s hand when he performed an interactive request toward the participants. From a neurophysiological point of view, the more participants felt involved in the interaction, the lower was motor preparation in the muscle potentially needed to fulfill the actor’s request. We contend that social contexts are more likely to elicit motor preparation compared to non-social ones, and that muscular inhibition is a necessary mechanism in order to prevent unwanted overt reactions during action observation tasks.

## Introduction

When interacting with another agent, gaze direction represents key information for social communication [1,2]. Since childhood, people have the tendency to attend to an interacting agent’s gaze [3–6]. Gaze direction, in fact, induces reflexive shifts of attention in the onlooker [7–9], provides information regarding where and to what one is paying attention [10], and it may activate ‘joint attention’ between two agents [11]. In gaze cuing paradigms, participants are typically faster at detecting or identifying an object when it appears in an observed gaze’s direction, compared to when it is presented in the opposite side (for reviews see [12,13]).

Of relevance for the present study, is the evidence that the gaze of another person provides information related to her subsequent behaviors, in particular to her intentions to act upon objects [14,15]. It has been demonstrated that observing an agent simply gazing at an object elicits behavioral and neural responses similar to when the same agent performs a grasping action toward the very same object [16,17]. Seemingly, observing someone’s gaze has the ability to elicit in the onlooker a grasp representation, which is related to possible hand-object interactions [18].

These findings have been interpreted in terms of a ‘direct matching’ mechanism mediated by mirror neurons (MNs). MNs show comparable activation during both action execution and observation [19] and their activity is modulated according to an observed agent’s gaze direction [20]. These latter results demonstrate that gaze direction is coded by the MNs and confirm previous evidence that action and gaze are tightly linked at a neural level [21].

Although the contribution of gaze cues in modulating motor behavior during action observation has been established [22,23], the investigation of this phenomenon in a realistic social situation is still rare [24]. To our knowledge, the effect of another’s gaze direction on motor preparation and readiness to interact–a disposition to engage in socially meaningful situations [25]–has yet to be tested.

To fill this gap, in the present study we capitalized on an established paradigm for inducing complementary activations in the observers’ muscles (i.e., motor patterns completing each other’s mutual action according to a common aim; [26]) to investigate the role of gaze direction in a social interactive context from both a behavioral and a neurophysiological perspective. In particular, we focused on the congruency between upper limb movement direction and gaze direction during observation of video clips expressing a social request (i.e., interactive request action) or not (i.e., non-interactive action). Specifically, gaze and limb movement cues could both point toward the same object (i.e., convergent conditions) or gaze could point toward an object and limb movements toward a different one (i.e., divergent condition).

In Experiment 1, the location and duration of participants’ ocular fixations were investigated by means of eye-tracking procedures. This provided a measure of the relative influence of the coupling/uncoupling of gaze and action cues on observer’s gaze behavior, and their ability to attract overt attention to critical aspects of the visual scene. By dissociating gaze and movement cues in a social interactive context, we expect to disentangle their relative influence. In particular, we expect that eye-tracking data will provide vital information regarding whether and how the reading of others’ gaze and body movements is crucial for understanding the intention to interact.

In Experiment 2, we tested onlooker’s motor preparation via transcranial magnetic stimulation (TMS) paired with electromyography (EMG) during observation of the same video clips. Then we measured and correlated these neurophysiological data with the subjective involvement in the observed scene, acquired through a questionnaire. To the best of our knowledge, how the interplay between gaze and body cues influences corticospinal excitability in a context calling for a social involvement has never been tested. Based on previous results obtained using similar paradigms for inducing complementary activations in the observers’ muscles [26], we predict that coupling gaze direction with a request gesture will increase response saliency, therefore enhancing observers’ motor preparation and readiness to interact (see [27] for a similar approach). In particular, an increased activity of the muscle involved in the response preparation for the Interactive condition when the gaze points to the salient object is expected.

## Experiment 1

Observers’ eye movements were recorded during the observation of video clips showing interactive and non-interactive actions. We manipulated both the actor’s gaze and upper limb’s direction so that they could be convergent or divergent, and we sought to reveal how this manipulation affected the observer’s gaze behavior through eye-tracking procedures.

### Methods

#### Participants

**Fig 1:**
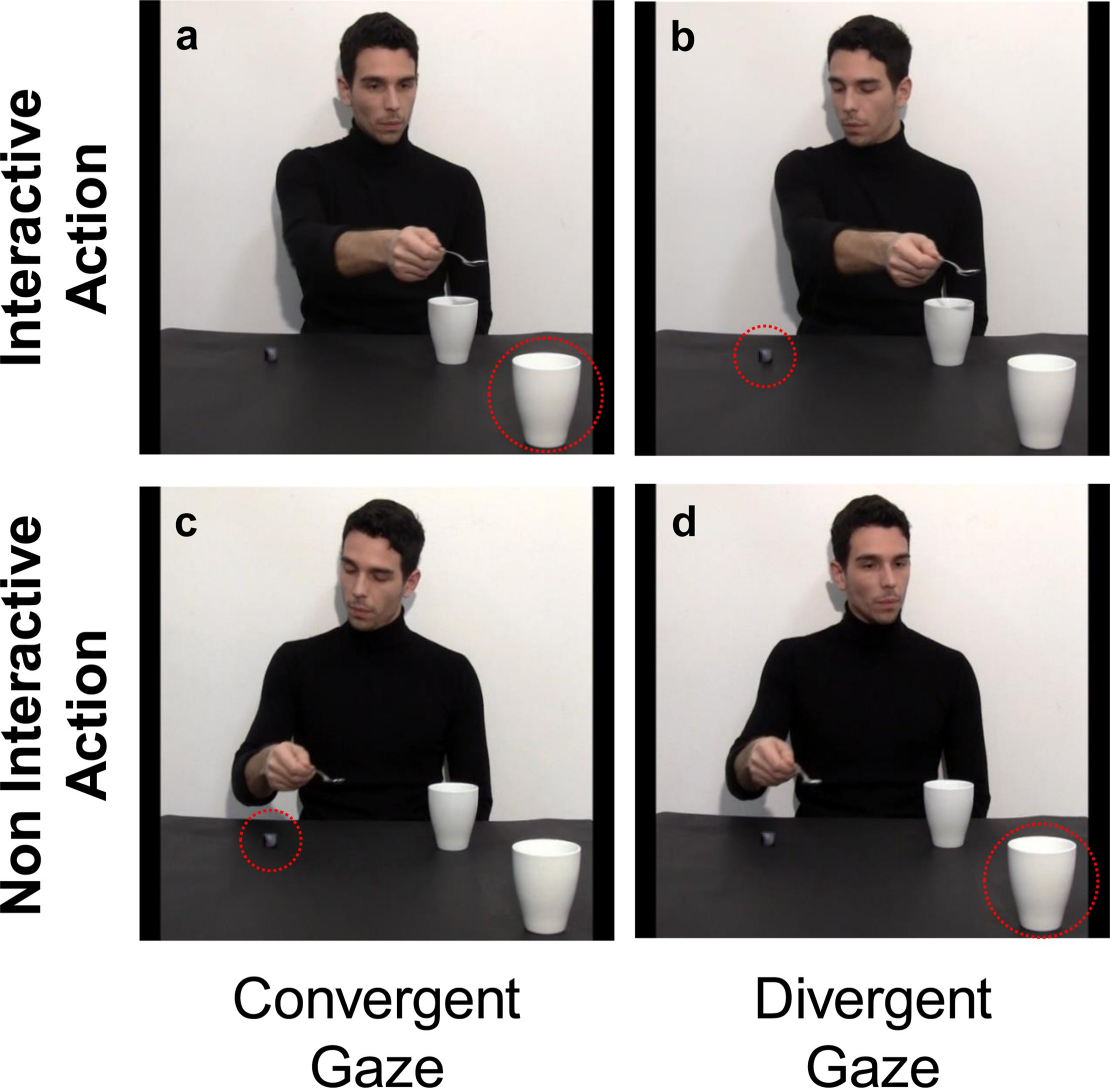
Each row represents an action: ‘Interactive request’ at top and ‘Non Interactive’ at bottom. Participant’s ocular fixations in the visual scene were manipulated by the actor’s gaze direction: ‘Convergent gaze’ (see left column) when the actor’s gaze and gesture were convergent; ‘Divergent gaze’ (see right column) when they were divergent. Red circles indicate the object to which the actor’s gaze was directed.

**Fig 2:**
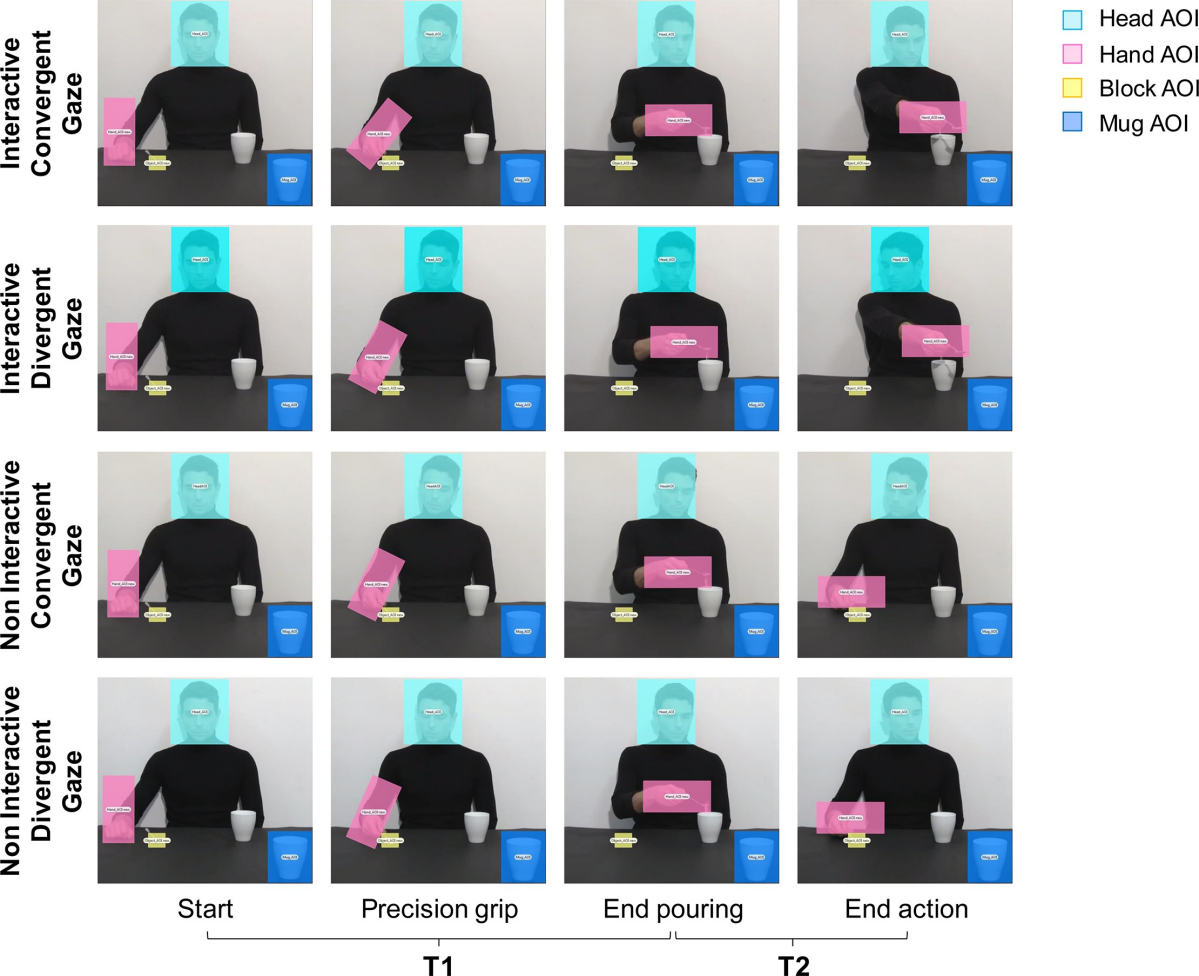
Sequence of events for the four experimental videos (i.e., Interactive request and Non Interactive Actions with Converging/Diverging Gaze directions) and the time epochs considered for data analysis (i.e., T1, T2). The overlaid colored rectangular areas represent the adopted AOIs: Head (light blue); Hand (pink); Block (yellow) and Mug (blue).

**Fig 3:**
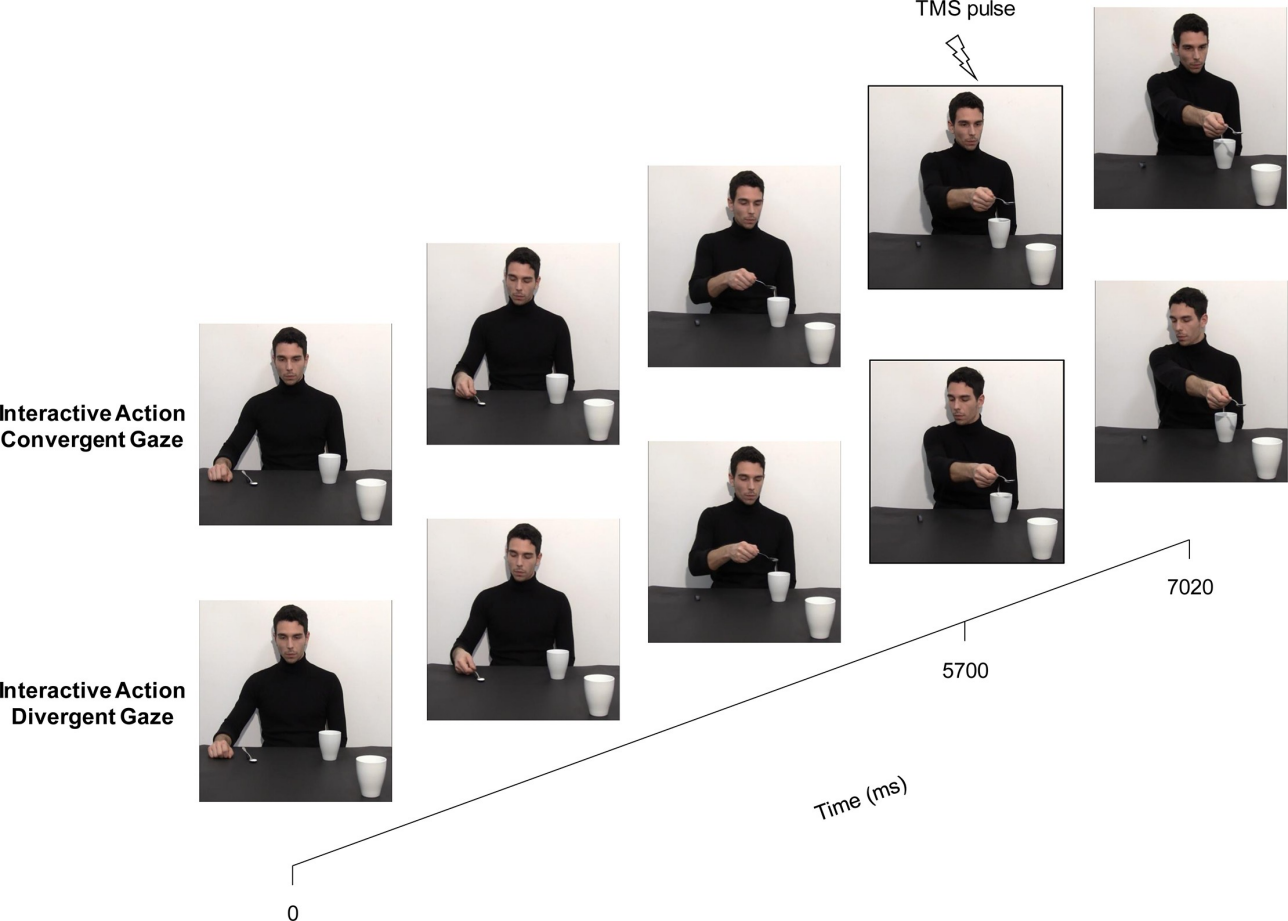
Timing of TMS stimulation was set at 5700 ms for all conditions. This time point was set at the starting of the second action step: reaching for the mug in the Interactive condition with Convergent/Divergent Gaze (see examples) or coming back to the starting block in the Non Interactive condition.

Twenty right-handed volunteers (15 females and 5 males, age range 21–31 years, mean age 24.8 years) with normal or corrected-to-normal vision took part in the experiment. They all provided written informed consent prior to the experiment. Ethical approval for conducting the experiment was granted by the University of Padua’s ethics committee, in accordance with the Declaration of Helsinki. A right-handed non-professional actor (male, 29 years old; pictured in Figs 1, 2 and 3) was recruited for video-clip recording. He provided written informed consent (as outlined in PLOS consent form) to publish his image alongside the manuscript.

#### Stimuli

Four types of actions were digitally recorded as experimental stimuli:

- *Interactive request action*, *Convergent Gaze*: an actor grasped a sugar spoon placed on a small starting block, poured some sugar into a mug placed next to him on a table, then, with some sugar left in his spoon, stretched out his arm toward a mug out of his reach–but strategically placed near the observer, as to require her intervention to lift it. Notably, the actor performed this request gesture while looking at the mug (Fig 1A).
- *Interactive request action*, *Divergent Gaze*: the actor performed the very same request gesture but while looking at the starting block instead of the mug (Fig 1B).
- *Non Interactive action*, *Convergent Gaze*: the actor grasped the sugar spoon, poured some sugar into the mug placed next to him and then he moved the spoon back to its initial position while looking at it (Fig 1C).
- *Non Interactive action*, *Divergent Gaze*: the actor performed the very same action, but when moving the spoon back to its initial position he looked at the mug (Fig 1D).

Note that the actor grasped the sugar spoon with his right hand using a precision grip (PG; i.e., opposition of thumb to index finger) and the same grasp was elicited by the small starting block. Whereas the other object present in the scene (i.e., the mug) required the use of a whole hand grasp (WHG; i.e., opposition of fingers to palm) to be handled. Therefore, the observed movement (i.e., PG) was specifically mismatched with the one required to interact in a complementary fashion (i.e., WHG) and both targets of the actor’s gaze were mismatched in terms of affordances. Each video lasted 7020 ms and the animation effect was obtained by presenting a series of single frames (40 ms duration) following the first frame lasting 500 ms.

#### Procedures

Participants were seated 65 cm from a monitor (1280 x 1024 pixels) and they were asked to observe the video stimuli (AVI format videos, 25 frames per second). Each trial started with a fixation cross presentation in the center of the monitor and participants were instructed to look at it for three seconds. This ensured all participants started observing the video stimuli from the same origin point. Each video clip was randomly presented three times to each participant. The experimental session lasted approximately ten minutes.

#### Eye tracking recordings

Eye movements were recorded by means of an infrared T120 Eye Tracker embedded in a 17” display (Tobii Technology, Danderyd, Sweden). Eye position was sampled at 120 Hz with a spatial accuracy of 0.5 degrees of visual angle. Prior to starting the experiment, the eye-tracker calibration was performed through a standard five-point grid, and repeated when necessary.

#### Eye-tracking analysis

Areas of Interest (AOIs) were created to investigate fixations targeted to specific regions using Tobii Studio 3.1 software. A fixation event was computed when gaze remained within 0.5 degrees of visual angle for at least 100 ms. For each video, four AOIs were adopted (see Fig 2): a) Head AOI (200 x 225 pixels): a static area which included the actor’s head; b) Hand AOI (109 x 233 pixels): a dynamic area which included the actor’s hand manipulating the sugar spoon; c) Block AOI (58 x 49 pixels): a static area including the starting block; and d) Mug AOI (151 x 177 pixels): a static area covering the mug placed near the observer, in the right corner of the screen. Participants’ gaze behavior within the AOIs was tracked for the entire duration of the video stimuli. The total Fixation Duration (i.e., the whole duration in seconds for all fixations within the selected AOI) was considered for gaze data analysis. Since the two action sequences were identical during the first action step (i.e., pouring sugar in the close mug), a difference in gaze parameters was expected only in the last part of the action, namely when the actor’s gaze direction was converging/diverging from the action’s direction. Data analysis has therefore been segmented into two epochs (Fig 2): i) T1, time between the start of the action and the end of pouring in the first mug (5000 ms); ii) T2, time between the end of pouring and the end of the action, that is, the actor’s arm extension toward the observer for the Interactive condition or the actor returning the spoon to the small starting block for the Non Interactive condition (2000 ms). Data were analyzed by means of a linear mixed-effects model, using as response variable each level (T1 and T2) of the Fixation Duration index. The various levels of AOI (Head, Hand, Block and Mug), Action (Interactive, Non Interactive) and Gaze direction (Convergent, Divergent) were used as predictors. The three predictors were used as fixed effects of the model and their interaction was inserted in the model. The individual variability was assessed setting participants as a random factor (random intercept model). Since the four AOIs differed in their dimensions, they were considered separately in the analysis to allow meaningful comparisons. The mixed model was performed using the nmle package in R [28]. Conditional R squared has been computed as a measure of goodness of fit of the tested models [29] by means of the MuMin R package [30]. A significance threshold of *p* < 0.05 was set for all statistical analysis. Each time a statistically significant effect was found, multiple comparisons were performed using the emmeans package [31]. Degrees of freedom of such comparisons were computed using the Satterthwaite method, while p-values were adjusted using the Tukey method [31,32]. Cohen’s *d* were computed for each multiple comparisons according to the method explained by Westfall, Kenny, and Judd [33].

### Results and discussion

During the first part of the action sequence (T1) only a significant main effect of AOI emerged, F_(3,285)_ = 107.54, *p* < 0.001 (Table 1). Participants observed longer the Head AOI, followed by the Hand, the Block and the Mug AOIs. At T2, a significant effect of the AOI predictor, F_(3,285)_ = 86.33, *p* < 0.001, as well as of the interaction between AOI and Action, F_(3,285)_ = 9.93, *p* < 0.001, AOI and Gaze direction, F_(3,285)_ = 6.94, *p* < 0.01, and the interaction among all the predictors, F_(3,285)_ = 5.80, *p* < 0.01, emerged, as displayed in Table 1. See S1 Table with descriptive statistics for all the three predictors (AOI, Action and Gaze direction) for each time epoch (T1, T2).

**Table 1:** Mixed model effects for the Fixation Duration variable for T1 and T2.

	FIXATION DURATION			
	T1		T2	
	F (Df)	P-value	F (Df)	P-value
**AOI**	107.54 (3.285)	<0.001*	86.33 (3.285)	<0.001*
**Condition**	0.03 (1.285)	0.87	0.01 (1.285)	0.91
**Gaze**	0.28 (1.285)	0.60	0.001 (1.285)	0.97
**AOI x Condition**	1.32 (3.285)	0.27	9.93 (3.285)	< 0.001*
**AOI x Gaze**	0.85 (3.285)	0.47	6.95 (3.285)	< 0.01*
**Condition x Gaze**	0.62 (1.285)	0.43	2.11 (1.285)	0.15
**AOI x Condition x Gaze**	0.11 (3.285)	0.95	5.80 (3.285)	< 0.01*
	**Conditional R**^**2**^ **= 0.52**		**Conditional R**^**2**^ **= 0.51**	

F statistics, degrees of freedom (Df) and p-values are presented. Asterisks indicate statistically significant values (*p* < 0.05).

Fixation Duration results for each AOI at T2 are reported as follow:

*Head AOI*: Gaze presented a significant effect, F_(1,57)_ = 6.17, *p* < 0.001, with longer fixations when the actor’s gaze was pointing to the mug compared to when it was directed toward the starting block, *t*_(57)_ = -2.48, *p* = 0.016, d = 0.43. A significant interaction between Action and Gaze direction also emerged, F_(1,57)_ = 13.36, *p* < 0.001. Fixations were longer when hand and gaze direction were divergent. In particular, longer fixations were found for the Interactive Request Action, Divergent Gaze, *t*_(57)_ = -2.86, *p* < 0.001, d = 0.71, and the Non Interactive Action, Divergent Gaze, *t*_(57)_ = -4.34, *p* < 0.001 d = 1.07, conditions compared to the Non Interactive Action, Convergent Gaze condition.

*Hand AOI*: A significant effect of Action emerged, F_(1,57)_ = 15.41, *p* < 0.001. Participants spent more time watching the actor’s hand in the Interactive compared to the Non Interactive action sequence, *t*_(57)_ = -3.92, *p* < 0.001, d = 0.80.

*Block AOI*: A significant effect of both Action, F_(1,57)_ = 26.97, *p* < 0.001, and Gaze direction, F_(1,57)_ = 19.38, *p* < 0.001, predictors emerged, together with the interaction among them, F_(1,57)_ = 6.50, *p* = 0.014. Specifically, in the Non Interactive condition participants looked longer at the small block when the actor’s action and gaze were both pointing to it (Convergent Gaze) compared to when gaze was directed away, *t*_(57)_ = 5.48, *p* < 0.001, d = 1.61. Fixations in the Non Interactive Action, Convergent Gaze condition were also longer than in the Interactive Action condition for both Divergent, *t*_(57)_ = 4.92, *p* < 0.001, d = 1.45, and Convergent Gaze directions, *t*_(57)_ = 6.78, *p* < 0.001, d = 1.99.

*Mug AOI*: when considering the salient object for the social interaction, a significant effect of Gaze direction emerged, F_(1,57)_ = 12.35, *p* < 0.001, with longer fixations when the right-oriented gaze was pointing toward it, compared to when the actor was looking at the opposite side, t_(57)_ = -3.52, *p* < 0.001, d = 0.66.

To sum up, eye tracking data show the direction of the actor’s gaze and gesture differently influenced the onlookers’ observation behavior. In particular, the actor’s head was attended more when his gaze and gesture were divergent, thus suggesting the adoption of a strategy for disambiguating the action’s outcome and understanding the actor’s intention [34]. In this line, participants fixated longer the salient object (i.e., the mug) when the actor’s gaze was directed toward it, regardless of his action’s direction, thus highlighting the specific role of gaze cues for intention reading in social contexts. The actor’s hand was more attended when expressing a social request toward the observer, regardless of gaze direction. Only the small starting block was more attended when the two indexes (i.e., gaze direction and hand gesture) were jointly directed at it. It seems therefore that different cues have different weight depending on context. Social gestures toward the participants attract their attention onto hand movements, whereas gaze allows disambiguating other’s intentions and specifically emphasizes socially-salient objects. Experiment 2 was then designed to explore how gaze and action cues differently affect observers’ motor preparation in hand muscles and readiness to interact.

## Experiment 2

Participants’ corticospinal excitability was measured during observation of the action sequences adopted in Experiment 1. If coupling gaze direction and request gesture enhances response saliency, then an increase in observers’ motor preparation and readiness to interact is expected. In particular, an increase of the MEP amplitude is expected for the muscle involved in the response preparation (i.e., the ADM) for the Interactive request condition with the gaze pointing to the mug.

### Methods

#### Participants

Thirty naïve volunteers (17 female and 13 male, aged between 19 and 28 years, mean age 21.8 years) with the same characteristics as those who participated in Experiment 1 took part in the experiment. They were all screened for TMS exclusion criteria and for neurological, psychiatric and medical problems [35,36]. The study was approved by the ethics committee of the University of Padua, in accordance with the Declaration of Helsinki. All participants gave their written informed consent and were financially compensated for their participation.

#### Experimental stimuli

The same experimental stimuli as for Experiment 1 were used (see Fig 1).

#### Procedure

Participants were tested in a single experimental session lasting approximately one hour. They were seated in a comfortable armchair with their right arm positioned on a pillow and their head on a fixed head rest. They were instructed to remain as still and relaxed as possible while watching video clips presented on a 24” monitor (resolution 1920 x 1080 pixels, refresh frequency 120 Hz) set at eye level (eye-screen distance was 80 cm). No specific task was given to participants; however, to ensure attention to the video-clips, they were told that at the experiment’s end they would be questioned about the stimuli presented (post-experiment questionnaire). TMS-induced motor evoked potentials (MEPs) were acquired from the participants’ first dorsal interosseous (FDI) and abductor digiti minimi (ADM) muscles of their right hand. A single TMS pulse was released during each video presentation at 5700 ms, namely when the actor was starting to stretch out toward the out-of-reach mug (Interactive conditions; Fig 1A and 1B; Fig 3) or when he was returning the sugar spoon to its initial position on the small starting block (Non Interactive conditions; Fig 1C and 1D; Fig 3). Notably, the actor was also directing his gaze leftward or rightward at this time point (Fig 3). The order of the video-clips was randomized across participants. A total of 120 MEPs (2 muscles x 2 actions x 2 gaze directions x 15 repetitions) were recorded for each participant. Prior to and after the experimental block, each participant’s baseline corticospinal excitability was assessed by acquiring 15 MEPs while they passively watched on the computer screen a white fixation cross on a black background (10 s inter-pulse interval: for 5 s the remainder to remain fully relaxed was showed, followed by other 5 s of fixation cross presentation). Possible variations in corticospinal excitability related to TMS per se were assessed by comparing the MEP amplitudes recorded during the two baseline periods (30 MEPs in total). Their average amplitude was then utilized to set each participant’s individual baseline for data normalization procedures. Stimuli presentation, timing of TMS stimulation and EMG recordings were managed by E-Prime v2.0 software (Psychology Software Tools Inc., Pittsburgh, PA, USA) running on a computer.

#### TMS and EMG recording

Single-pulse TMS was administered to the hand region of the left primary motor cortex (M1) using a 70 mm figure-of-eight coil connected to a Magstim Bistim2 stimulator (Magstim Co., Whitland, UK). The coil was placed tangentially on the scalp, with the handle pointing laterally and caudally [37,38], in correspondence with the optimal scalp position (OSP) where MEPs with maximal amplitude were recorded simultaneously from the FDI and ADM muscles with the minimum stimulation intensity. These muscles were chosen as ADM is specifically involved during WHG but not PG, whereas FDI is more activated for PG than WHG [39]. To find the individual OSP, the coil was moved in steps of 0.5 cm until the position was reached. Once the OSP was found, it was marked on a tight-fitting cap worn by the participant and the individual resting motor threshold (rMT) was determined. The rMT is defined as the lowest stimulus intensity at which TMS is able to generate MEPs of at least 50 μV in relaxed muscles in 5 out of 10 consecutive pulses [40]. The stimulation intensity was then set at 120% of the rMT to record a clear and stable MEP signal throughout the experiment. rMT ranged from 30 to 56% (mean = 41%, SD = 6.2) of the maximum stimulator output. During the experimental sessions the coil was held by a tripod and continuously checked by the experimenters to maintain a constant positioning with respect to the marked OSP.

The EMG signal was recorded by means of two pairs of surface Ag/AgCl electrodes (1 cm diameter) placed in a belly-tendon montage, with the active electrode placed over the muscle belly and the reference over the interphalangeal joint. The ground electrode was positioned over the participant’s left wrist. Skin impedance, evaluated at rest prior to beginning the experimental session, was considered of good quality when below the threshold level (5 Ohm). Electrodes were connected to an isolable portable ExG input box linked to the main EMG amplifier for signal transmission via a twin fiber optic cable (Professional BrainAmp ExG MR, Munich, Germany). The raw myographic signals were band-pass filtered (20 Hz—1 kHz), amplified prior to being digitalized (5 KHz sampling rate), and stored on a computer for off-line analysis. Trials in which any EMG activity greater than 100 μV was present in the 100 ms window preceding the TMS pulse were discarded to prevent contamination of MEP measurements by background EMG activity. EMG data were collected for 300 ms after the TMS pulse by using Brain Vision Recorder software (Brain Products GmbH, Munich, Germany).

#### Post-experimental questionnaire

At the end of the experimental session, to quantify the subjective experience of involvement experienced by each participant for each experimental stimulus, they were asked to rate four sentences for each video on a five-point Likert scale (ranging from ‘Not at all’ to ‘Very much’). The order of the videos was counterbalanced across participants. The four sentences adopted were: Q1) “I felt involved in the action”; Q2) “At the end of the video I looked at where the actor was gazing at”; Q3) “At the end of the video the actor’s gaze distracted me from the action”; Q4) “At the end of the video I would have grabbed the nearest mug”. The relationship between the participants’ perceived level of engagement (low vs. high) and the corresponding CE modulations during video observation was a key factor of our study. We then clustered our participants in two groups of responders (i.e., Low and High Responders) to measure whether low or high scores to the questionnaire’s items were associated with different patterns of motor activations (see [27] for a similar approach).

#### Data analysis

**Mixed-effects model**

The MEP peak-to-peak amplitudes for FDI and ADM muscles were determined as a measure of participants’ corticospinal excitability. For each participant and experimental condition, MEPs were normalized computing a ratio between MEP amplitude values recorded during the experimental conditions and during the baseline blocks. As for Experiment 1, a linear mixed-effects model was used to assess the effects of Action (Interactive, Non Interactive), Gaze direction (Convergent, Divergent) and Muscle (FDI, ADM) on normalized MEP scores, setting participants as a random factor. Cohen’s *d* were computed for each multiple comparisons [33].

**A priori comparisons**

Data analysis then focused on a priori comparisons on the crossed level of condition and gaze predictors to explore possible differences between MEP scores collected in the Interactive condition with a right-oriented gaze and all the other crossed levels (treatment-contrasts method).

**Spearman’s rank-order correlations**

Spearman’s rank-order correlations were tested on MEP scores for both levels of the Muscle predictor and each of the four questions referring to each experimental condition. The aim was to explore a possible link between the perceived engagement with the observed action and corticospinal activity. Each p-value obtained was corrected with Bonferroni correction.

**Cluster analysis**

A k-means cluster analysis was performed on the questionnaire responses. Two centroids for the cluster membership configuration were set in order to create two theoretical groups of responders: Low and High Responders. These two groups were then used as a between factor for an ANOVA, and post-hoc tests were performed to assess if there were any differences in MEP scores between groups.

### Results

To control for nonspecific changes in corticospinal excitability that could have biased the results, for each muscle we compared the mean raw MEP amplitudes recorded at the beginning and at the end of the experimental session (baselines). No significant differences for either the FDI, *t*_(29)_ = 0.946, *p* = 0.352, or the ADM, *t*_(29)_ = 0.086, *p* = 0.932, muscles emerged.

#### Mixed-effects model

The Muscle predictor presented a significant effect on the MEP scores, F_(1,203)_ = 40.19, *p* < 0.001, FDI muscle had greater MEP values than ADM, *t*_(203)_ = -6.34, *p* < 0.001, d = 0. 66 (Table 2). The Gaze predictor also showed a significant effect on MEP scores, F_(1,203)_ = 6.29, *p* = 0.013 (Table 2). MEP values for the conditions in which the actor was looking at the mug were significantly lower than those for the conditions in which the actor was looking at the starting block, *t*_(203)_ = 2.5, *p* = 0.013, d = 0.26. The Action predictor and all the interactions among predictors did not reach a significant effect on the MEP scores.

**Table 2:** Results of the mixed-effects model.

	F value	P value
**Muscle**	40.190	<0.001*
**Action**	0.090	0.765
**Gaze direction**	6.294	0.013*
**Muscle x Action**	0.343	0.559
**Muscle x Gaze direction**	0.432	0.512
**Action x Gaze direction**	2.269	0.134
**Muscle x Action x Gaze direction**	0.026	0.872
**Conditional R^2^ = 0.637**		

Asterisks indicate statistically significant values (p < 0.05).

#### A priori comparisons

**Fig 4:**
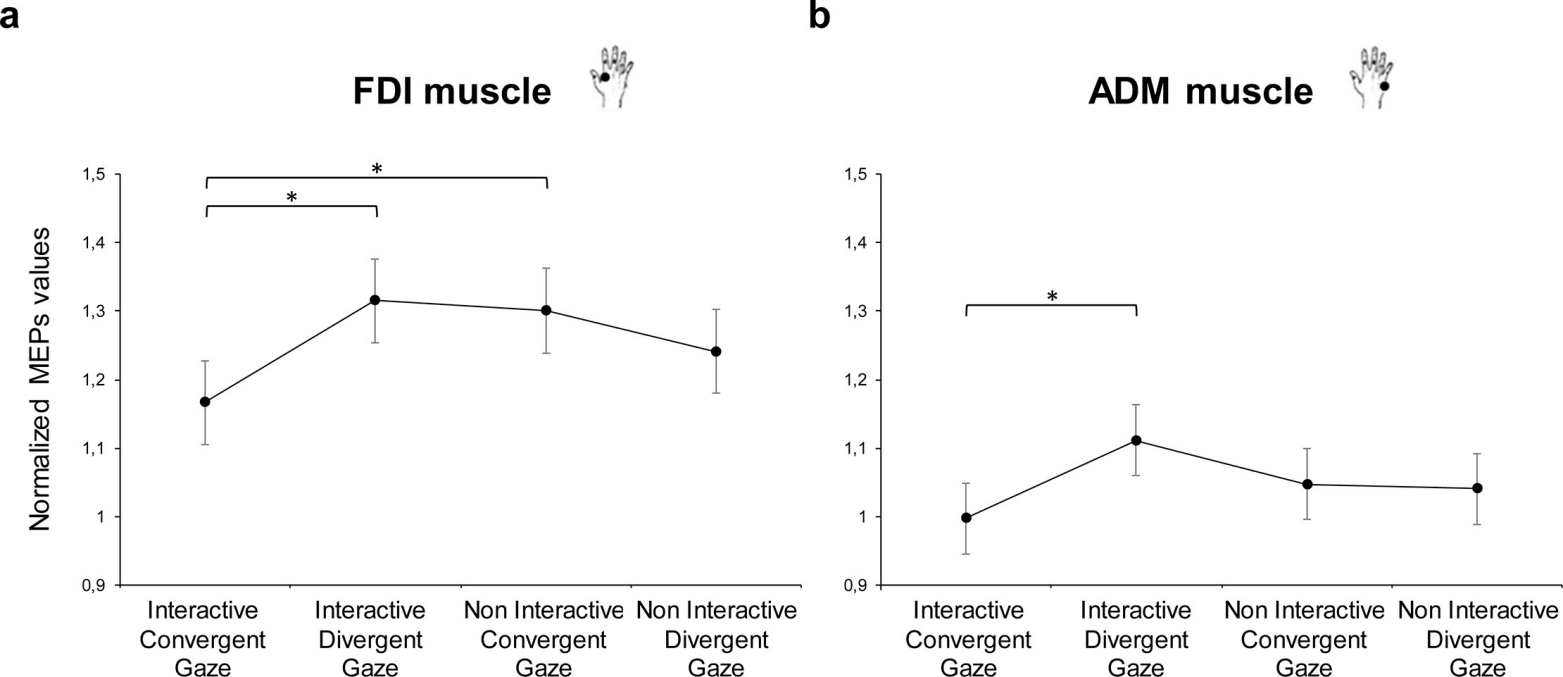
Planned contrasts on the crossed levels of the Condition and Gaze predictors. Bars refer to the standard error. Asterisks indicate statistically significant comparisons (p < 0.05).

A priori multiple comparisons on the crossed levels of the Action and Gaze predictors showed that FDI MEP values were higher when the actor’s gaze was pointing to the small starting block for both the Interactive request, *t*_(87)_ = 2.4, *p* = 0.019, d = 0.41, and the Non Interactive, *t*_(87)_ = 2.15, *p* = 0.034, d = 0.37, Action conditions, compared to when it was directed to the mug in the Interactive request Action, Convergent Gaze condition (Fig 4A). No difference instead emerged when comparing the Interactive request and the Non Interactive actions when the gaze was pointing to the mug (Convergent and Divergent Gaze, respectively), *t*_(87)_ = 1.2, *p* = 0.239, d = 0.2. Concerning the ADM muscle, MEPs for the Interactive, Convergent Gaze condition were lower than for the Interactive, Divergent Gaze condition, *t*_(87)_ = 2.22, *p* = 0.029, d = 0.36 (Fig 4B). No other significant differences emerged for the ADM muscle (p_s_ > 0.33).

#### Spearman’s rank-order correlations

**Fig 5:**
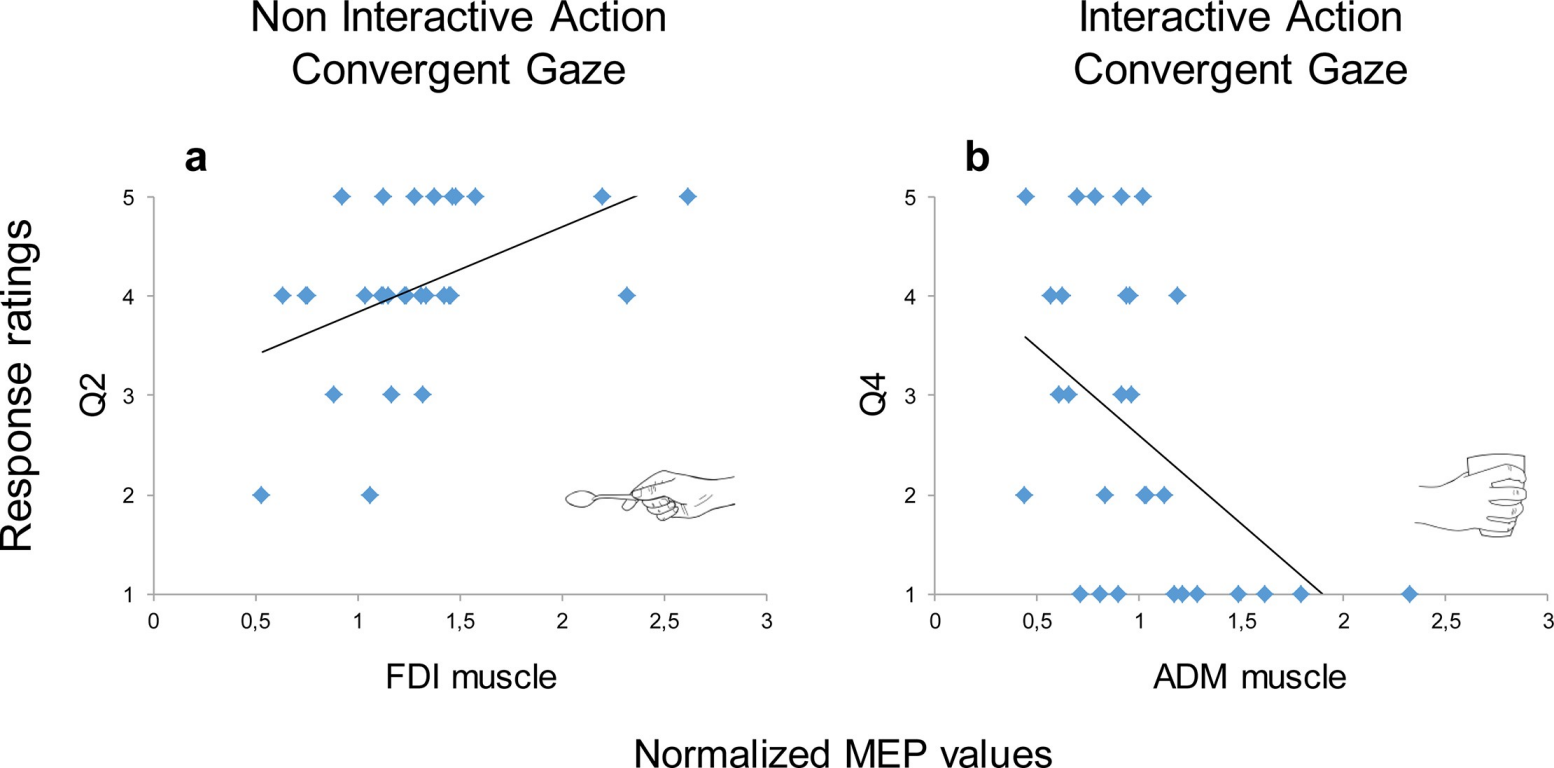
Results showing the statistically significant correlations between the response rating to the questionnaire statements (y-axis) and the normalized MEP values (x-axis). a) The FDI muscle activity in the Non Interactive condition Convergent Gaze was positively correlated with the questionnaire responses to the question 2 (Q2); b) the ADM muscle activity in the Interactive condition with Convergent Gaze was negatively correlated with the questionnaire responses to question 4 (Q4).

The Spearman’s rank-order correlation coefficient was used to assess the relationship between responses to each of the four questions and the MEP values for each muscle and experimental condition. Among the 32 computed correlations, only two reached statistical significance once Bonferroni correction was applied (Fig 5).

As concerns the Non Interactive Action, Convergent Gaze condition, results showed a positive correlation between the responses to the second statement of the questionnaire (Q2): “At the end of the video I looked at where the actor was gazing at” and the FDI muscle activity, r_s(28)_ = 0.52, *p* = 0.013 (Fig 5A). So, when gaze direction was convergent with the observed action (i.e., the actor gazed at the small block while moving back the sugar spoon toward it), an increase in the MEP amplitudes for FDI muscle was correlated with the perception of being captured by the actor’s gaze. The ADM MEP amplitude for the Interactive condition when the gaze was pointing to the mug was instead negatively correlated with the responses to the fourth statement of the questionnaire (Q4): “At the end of the video I would have grabbed the nearest mug”, r_s(28)_ = -0.47; *p* = 0.036 (Fig 5C). The more the participants were willing to respond to the action, the lower was their MEP amplitude in the muscle specifically required to perform the WHG. The subjective evaluation regarding the willingness to act toward the mug was actually correlated with a decrease of motor response in the observers’ muscle.

#### Cluster analysis

In order to assess two potential groups of responders, namely Low and High Responders, two clusters were set a priori based on the questionnaire results. This configuration has been adopted for each level of the Action predictor, crossed with both levels of the Gaze predictor. All the clusters are shown in Table 3.

**Table 3:** Cluster analysis.

	INTERACTIVE CONVERGENT GAZE		INTERACTIVE DIVERGENT GAZE		NON INTERACTIVE CONVERGENT GAZE		NON INTERACTIVE DIVERGENT GAZE	
	Low	High	Low	High	Low	High	Low	High
**Q1** «I felt involved in the action»	2.7 (1.05)	4.07 (0.76)	2.57 (1.16)	2.87 (1.02)	1.66 (0.79)	3.55 (0.73)	2.13 (0.64)	3.5 (0.52)
**Q2** «At the end of the video I looked at where the actor was gazing at»	4 (0.93)	4.3 (0.85)	3 (1.51)	4.31 (0.48)	3.95 (0.86)	4.44 (0.73)	4 (0.76)	3.87 (0.88)
**Q3** «At the end of the video the actor’s gaze distracted me from the action»	2.41 (1.17)	2.77 (1.01)	1.64 (0.63)	3.87 (0.88)	2.38 (1.07)	2.77 (1.3)	2.07 (0.7)	3.67 (0.97)
**Q4** «At the end of the video I would have grabbed the nearest mug»	1.41 (0.61)	4.15 (0.8)	1.71 (1.13)	2.75 (1.24)	1.38 (0.59)	2.77 (1.2)	1.53 (0,64)	2.53 (1.5)

For each condition, the mean responses to the questionnaire items and standard deviations (in brackets) are reported for both Low and High Responders.

The cluster of the High Responders in the Interactive Action, Convergent Gaze condition was composed by participants who felt very engaged in the displayed action: in fact, they tended to assume the same gaze orientation of the actor and they would have grabbed the nearest mug at the end of the video. The two clusters presented a significant difference in MEP scores within the ADM level of the Muscle predictor, FDI: F_(1,28)_ = 2.298, *p* = 0.14; ADM: F_(1,28)_ = 6.198, *p* = 0.02 (Table 4), where High Responders showed lower MEP scores than Low Responders, *t*_(23.86)_ = -2.72, *p* = 0.012, d = 0.91. The more participants felt involved in the action and were willing to respond to it in a complementary fashion, the lower was the motor response in the ADM muscle (i.e., the muscle potentially involved in grabbing the mug). No differences emerged between Low and High Responders in the other conditions (see Table 4).

**Table 4:** Comparisons between Low and High Responders for each muscle and condition.

Low vs. High Responders	FDI		ADM	
	F_(1,28)_	P value	F_(1,28)_	P value
**Interactive Convergent Gaze**	2.30	0.14	6.20	0.02*
**Interactive Divergent Gaze**	0.02	0.89	2.07	0.17
**Non Interactive Convergent Gaze**	0.49	0.49	0.09	0.78
**Non Interactive Divergent Gaze**	0.07	0.80	0.57	0.46

Comparisons between Low and High Responders for each Muscle level (FDI, ADM) and condition. Asterisk indicate statistically significant comparisons (p < 0.05).

## Discussion

The present research aims at disentangling the role of other’s gaze and body cues in a social context evoking a motor response preparation in the onlooker (i.e., a complementary response) from both a behavioral and a neurophysiological perspective. Observers’ eye movements, corticospinal excitability and readiness to interact were measured while an actor’s gaze and upper limb were shown either in a convergent or divergent direction. In the interactive condition, an actor was shown moving his hand toward a socially-salient object that was clearly out of her reach, but that was near to the observer’s peripersonal space–in fact, a social response implicating grasping the object was triggered in the observer. First and foremost, we found that the socially-salient object was specifically attended when the actor’s gaze was directed toward it, regardless of his action (interactive/non interactive). Moreover, divergent cues provided by the actor’s gaze and upper limb movements attracted participants’ overt attention to his head, as a strategy to disambiguate the scene and detect his intention. The social request directed toward the participants, as expected, focalized their overt attention to the observed hand movement, regardless of gaze direction. These findings seem to suggest that gaze cues are crucial when the social nature of an observed context has to be defined and intentions are not yet deciphered. In order to investigate how our gaze and body cues manipulation was coded at the motor level, we tested corticospinal excitability of M1 and readiness to interact. Our results extend previous literature by showing the congruent combination of gaze and hand request increased observer’s readiness to interact. In motor terms, we found an increased activity in the observers’ FDI muscle, the same muscle involved in the observed action, when the actor’s gaze was pointing to the small starting block. In line with previous evidence, the increased excitability in the observers’ index finger muscle reflected an activation compatible with the actor’s action (i.e., direct matching) and this effect was enhanced when attention was drawn to the small object eliciting a compatible motor activation [41–43]. Interestingly, when both the hand request and gaze direction were converging to the object next to the observer, decreased ADM muscle activity was measured.

### The motor inhibition hypothesis

According to Naish and colleagues [44], an inhibitory pattern of activity can be interpreted as a mechanism preventing the tendency to react to an observed stimulus, when a response is not required. This might be particularly true for social stimuli. A real-time interactive study from Sartori and colleagues [45] demonstrated indeed that a social request directed to participants was able to activate a quick and uncontrollable complementary response, regardless the given instructions was to refrain from doing it. In the present experiment, participants were required to remain perfectly still and relaxed while watching the actions, so it is possible that a similar quick response preparation took place in the motor system, and inhibition was required to prevent overt behavior. Eye-tracking data and questionnaire results seem to exclude that the lack of motor facilitation reflects a lack of attention/interest toward the action or salient object. Inhibition can instead be interpreted as a ‘rebound effect’ [46,47], as reported by Schuch et al. [48] in an EEG study. These authors found that the more the mirror system was activated (i.e., greater desynchronization of the mu rhythm over sensorimotor cortex during action observation), the more the motor system was subsequently inhibited (i.e., greater synchronization of the mu rhythm [48]). Crucially, a connection between mu power and MEPs has been provided by some studies demonstrating that increased mu oscillations are associated with a reduction of MEP amplitude [49–51]. Overall, this evidence suggests that the specific inhibition registered at the ADM muscle level could have been a byproduct of an increased pre-activation. Along this line, we recently demonstrated that gaze engagement during a social request led to a greater activation of corticospinal excitability at an early stage rather than at the end of the gesture [27].

Further support to the hypothesis of a mechanism to prevent an overt response elicited by the salient request comes from the correlation between the perceived impulse to grasp the mug and the motor excitability measurements. The more the participants reported their willingness to grab the mug at the end of the action sequence, the more their corticospinal excitability was decreased in the muscle required to interact with it. The fact the corticospinal excitability was not consistent with participants’ subjective report is in line with a recent study, showing that the observers’ own intentions and motivations during an experiment are independent from motor excitability during action observation [52]. Furthermore, when considering Low and High Responders–who showed respectively little or high engagement to the observed action sequences–differences in ADM muscle activations were present. In the Interactive condition, when action and gaze were both pointing to the salient object, High Responders showed lower MEPs in their little finger muscle compared to Low Responders. Therefore, it is precisely in the subgroup of High Responders that muscular inhibition was greater.

Previous research demonstrated that, during action observation, the spinal cord excitability–tested by eliciting the H-reflex in finger muscles–varied in accordance with the observed movements, but in the opposite direction to that occurring when executing them [53]. It was then suggested that the emerged inhibitory pattern in the spinal cord could allow the motor system to replicate the observed action internally, while preventing its overt replica. Consistent with the discovery of ‘anti-mirror’ neurons in the human brain (i.e., neurons increasing their firing rate during action execution, but decreasing it during observation of the same action; [54]), it is possible our motor system contains mechanisms that, while allowing us to resonate with others’ actions, still ensure we do not imitate them or react to them when it is not required (see also monkey literature: [55,56]).

As a final issue, the fact we found a pattern of inhibitory activity, which is not always detected in action observation studies, could depend on the type of stimuli adopted. As suggested by Naish and colleagues [44], it is possible that ecological contexts are more likely to elicit motor preparation compared to others, and that these conditions consequently necessitate of a suppression mechanism [57]. According to their model, in order to prevent production of overt imitation of observed movements, inhibitory processes would follow an early increase of corticospinal excitability due to action observation. The authors propose that the inhibitory mechanism might occur either (or both) in parallel with excitatory processes, or be triggered when the excitation level reaches a certain threshold [44]. In this view, our results are in line with what the Naish and colleagues [44] model predicted.

### The role of attention in social motor preparation

From an attentional point of view, previous literature suggests that other’s gaze triggers fast and automatic shifting of attention toward the gazed location, as occurs with sudden onset cues [58]. A previous experiment in which attention to various parts of an interactive scene was manipulated by means of rapid dot presentation [41] showed direct matching is dependent on attentional allocation toward an actor’s hand, in line with previous literature [48,59–61]. However, motor preparation for a complementary action resulted unaffected by an attention-diverting cue [41]. Here, by manipulating attention orienting through gaze direction, we replicated the direct matching results in the Non Interactive context. Moreover, a decrease of corticospinal excitability emerged when gaze direction and a request gesture toward an object were coupled. It could be argued that the modulation of corticospinal excitability specifically found for this condition is due to simply having two convergent cues attracting attention toward the object, without the effect being intrinsically social. We ruled out this alternative explanation in a previous study, where a moving arrow pointing toward the object replaced the social gesture [62]. The results showed that the arrow determined a much lower MEP activation compared to the request gesture ([62], see also [63]). The results of the present study then extend available literature on complementary actions. In previous experiments, indeed, the ADM muscle resulted activated in response to a request gesture, but the actor’s head was not visually available in the scene [26]. Interestingly, having made the actor’s head and gaze visually available led to different results. Put simply, the observed gaze may have added ‘social noise’ to the visual scene. Bunday and colleagues [64] have recently demonstrated that the presentation of a whole person performing an action–compared to the presentation of only the acting hand–abolished grasp-specifics effects in motor resonance, without the results being due the relative size of the observed hand. Moreover, the available literature on attention manipulation during action observation was mainly confined to paradigms adopting simplified stimuli (e.g., a big hand over a neutral background) that, although boosting subtle responses in neural activity, may lack in their ecological validity [60,65].

### How gaze and body cues interplay influences social affordance

Affordances are action possibilities, associations between environmental properties and abilities [66,67]. It has been proved that these action possibilities can be modulated by the task, as well as by the physical and social contexts (e.g. [68–70]). Our environment is typically crowded with objects and people, and we usually perceive objects in presence of other people who show different intentions to act. Social inputs may influence affordance perception, modulating our motor responses. *Social affordances* specifically refer to affordances modulated by social situations and contexts [71]. They depend on the presence of social signals, whose function is to alter a recipient’s behavior by triggering a range of opportunities for actions [72,73], and the willingness to engage in an interactive task [74]. Our gaze and body manipulation was intended to differently affect affordance toward the objects in the visual scene. Critically, when the actor expressed the interactive request, this was meant to induce in the observer the tendency to grasp the salient object for the potential interaction. When action and gaze were both pointing toward the object, the social affordance activation would push the observer to grasp the object, even if no real interaction takes place (see [75]). Conversely, interference effect due to simultaneous activation of affordances for two different objects may have occurred when gaze and action cues were diverging. Interestingly, in the Interactive request action with Divergent gaze, an increase of both FDI (coding for PG on the observed small starting block) and ADM (coding for WHG on the mug to which the hand action was directed) muscles emerged, compared to the Convergent gaze condition in which no mismatch between action and gaze was shown. It could be argued that the increased activation in the Divergent condition could be due to the ‘oddness’ of gaze and action’s mismatch. However, divergent cues are quite frequent in deceiving and bluffing contexts (e.g., when a soccer player looks at the opposite side of a goal from the one in which he kicks the ball or when someone looks at us while stealing our wallet). Deception is quite a common behavior and the present results might offer new insights for the related literature (e.g., [76,77]).

In our study, the activation of social affordances as a consequence of observing the interactive request toward the mug, instead of an increase in muscle activation determined a decrease of its response. These results are in line with what Wokke and colleagues [78] found investigating the strength of the responsiveness to affordances in real environments. They showed that when the context strongly activates specific affordances greater (inhibitory) efforts are required if the responses have to be withheld.

### Towards an ecological approach to the study of social interactive behavior

In the present study, we adopted video-clips depicting realistic social situations and we studied observers’ motor modulations through multiple methods: eye-tracking, corticospinal excitability modulations and self-report responses. This approach represents a compromise between the use of a realistic social situation and the adoption of a controlled experimental design, as suggested by Readers and Holmes [79]. Nonetheless, we acknowledge that testing gaze and body interplay during *real* interactive situations is the new frontier [65]. The effort to design experiments that may help unveil the motor system functioning in realistic situations should be a challenge for future studies in this field. In addition, as suggested by Kingstone and colleagues [80] future research should ideally study phenomena as they occur in the real-world environment before being studied under controlled laboratory setting.

To conclude, even if future research is needed to explore the origins and the temporal aspects of the inhibitory activation that emerged in this set of data, this study demonstrates that the joint combination of gaze and action in a social interactive context increased participant readiness to interact, which resulted in an increased muscular inhibition. The higher was the tendency to interact, the higher was the inhibition in the required muscle. Overall, these data bring research on action observation and complementary actions a step forward, contributing to a better understanding of gaze and action’s roles in motor system functioning during realistic social situations.

## Supporting information

S1 Table Descriptive statistics for the fixation duration variable. Means and standard deviations (in brackets) for each time epoch (T1, T2), Action (Interactive, Non Interactive), Gaze direction (Convergent, Divergent) and AOI (Head, Hand, Block, Mug) are presented.
(DOCX)

## References

[1] Emery NJ. The eyes have it: the neuroethology, function and evolution of social gaze. Neurosci Biobehav Rev. 2000;24: 581–604. 10.1016/S0149-7634(00)00025-7 10940436

[2] Porciello G, Crostella F, Liuzza MT, Valentini E, Aglioti SM. rTMS-induced virtual lesion of the posterior parietal cortex (PPC) alters the control of reflexive shifts of social attention triggered by pointing hands. Neuropsychologia. 2014;59: 148–156. 10.1016/j.neuropsychologia.2014.04.017 24813151

[3] Conty L, Tijus C, Hugueville L, Coelho E, George N. Searching for asymmetries in the detection of gaze contact versus averted gaze under different head views: a behavioural study. Spat Vis. 2006;19: 529–545. 10.1163/156856806779194026 17278526

[4] Farroni T, Csibra G, Simion F, Johnson MH. Eye contact detection in humans from birth. Proc Natl Acad Sci. 2002;99: 9602–9605. 10.1073/pnas.152159999 12082186 PMC123187

[5] Langton SRH, Watt RJ, Bruce V. Do the eyes have it? Cues to the direction of social attention. Trends Cogn Sci. 2000;4: 50–59. 10.1016/S1364-6613(99)01436-9 10652522

[6] Maurer D, Salapatek P. Developmental changes in the scanning of faces by young infants. Child Dev. 1976;47: 523–527. 1269319

[7] Driver JI, Davis G, Ricciardelli P, Kidd P, Maxwell E, Baron-Cohen S. Gaze Perception Triggers Reflexive Visuospatial Orienting. Vis Cogn. 1999;6: 509–540. 10.1080/135062899394920

[8] Friesen CK, Kingstone A. The eyes have it! Reflexive orienting is triggered by nonpredictive gaze. Psychon Bull Rev. 1998;5: 490–495. 10.3758/BF03208827

[9] Langton SRH, Bruce V. Reflexive Visual Orienting in Response to the Social Attention of Others. Vis Cogn. 1999;6: 541–567. 10.1080/135062899394939

[10] Bukowski H, Hietanen JK, Samson D. From gaze cueing to perspective taking: Revisiting the claim that we automatically compute where or what other people are looking at. Vis Cogn. 2015;23: 1020–1042. 10.1080/13506285.2015.1132804 26924936 PMC4743615

[11] Butterworth G. The ontogeny and phylogeny of joint visual attention Natural theories of mind: Evolution, development and simulation of everyday mindreading. Cambridge, MA, US: Basil Blackwell; 1991 pp. 223–232.

[12] Atkinson MA, Simpson AA, Cole GG. Visual attention and action: How cueing, direct mapping, and social interactions drive orienting. Psychon Bull Rev. 2017; 1–21. 10.3758/s13423-016-1113-7 28808932

[13] Frischen A, Bayliss AP, Tipper SP. Gaze cueing of attention: Visual attention, social cognition, and individual differences. Psychol Bull. 2007;133: 694–724. 10.1037/0033-2909.133.4.694 17592962 PMC1950440

[14] Becchio C, Bertone C, Castiello U. How the gaze of others influences object processing. Trends Cogn Sci. 2008;12: 254–258. 10.1016/j.tics.2008.04.005 18555735

[15] Sartori L, Becchio C, Castiello U. Cues to intention: The role of movement information. Cognition. 2011;119: 242–252. 10.1016/j.cognition.2011.01.014 21349505

[16] Castiello U. Understanding other people’s actions: Intention and attention. J Exp Psychol Hum Percept Perform. 2003;29: 416–430. 10.1037/0096-1523.29.2.416 12760625

[17] Pierno AC, Becchio C, Wall MB, Smith AT, Turella L, Castiello U. When gaze turns into grasp. J Cogn Neurosci. 2006;18: 2130–2137. 10.1162/jocn.2006.18.12.2130 17129195

[18] Ramsey R, Cross ES, Hamilton AF de C. Predicting others’ actions via grasp and gaze: evidence for distinct brain networks. Psychol Res. 2012;76: 494–502. 10.1007/s00426-011-0393-9 22120203

[19] di Pellegrino G, Fadiga L, Fogassi L, Gallese V, Rizzolatti G. Understanding motor events: a neurophysiological study. Exp Brain Res. 1992;91: 176–180. 10.1007/bf00230027 1301372

[20] Coudé G, Festante F, Cilia A, Loiacono V, Bimbi M, Fogassi L, et al Mirror Neurons of Ventral Premotor Cortex Are Modulated by Social Cues Provided by Others’ Gaze. J Neurosci. 2016;36: 3145–3156. 10.1523/JNEUROSCI.3220-15.2016 26985026 PMC4792931

[21] Jellema T, Baker CI, Wicker B, Perrett DI. Neural representation for the perception of the intentionality of actions. Brain Cogn. 2000;44: 280–302. 10.1006/brcg.2000.1231 11041992

[22] Letesson C, Grade S, Edwards MG. Different but complementary roles of action and gaze in action observation priming: Insights from eye- and motion-tracking measures. Front Psychol. 2015;6 10.3389/fpsyg.2015.00006 25999886 PMC4419854

[23] Prinsen J, Bernaerts S, Wang Y, de Beukelaar TT, Cuypers K, Swinnen SP, et al Direct eye contact enhances mirroring of others’ movements: A transcranial magnetic stimulation study. Neuropsychologia. 2017;95: 111–118. 10.1016/j.neuropsychologia.2016.12.011 27939365

[24] Innocenti A, Stefani ED, Bernardi NF, Campione GC, Gentilucci M. Gaze Direction and Request Gesture in Social Interactions. PLOS ONE. 2012;7: e36390 10.1371/journal.pone.0036390 22693550 PMC3365068

[25] Di Paolo E, De Jaegher H. The interactive brain hypothesis. Front Hum Neurosci. 2012;6 10.3389/fnhum.2012.00006 22701412 PMC3369190

[26] Sartori L, Betti S. Complementary actions. Front Psychol. 2015;6: 557 10.3389/fpsyg.2015.00557 25983717 PMC4416362

[27] Betti S, Zani G, Granziol U, Guerra S, Castiello U, Sartori L. Look At Me: Early Gaze Engagement Enhances Corticospinal Excitability During Action Observation. Front Psychol. 2018;9 10.3389/fpsyg.2018.00009 30140243 PMC6095062

[28] Pinheiro J, Bates D, Debroy S, Sarkar D, R Core Team. nlme: Linear and Nonlinear Mixed Effects Models. R package version 3. 1–128. 2016; Available: http://CRAN.R-project.org/package=nlme

[29] Nakagawa S, Schielzeth H. A general and simple method for obtaining R2 from generalized linear mixed-effects models. Methods Ecol Evol. 2013; 133–142. 10.1111/j.2041-210x.2012.00261.x

[30] Bartoń K. MuMIn: Multi-Model Inference. 2018; Available: https://CRAN.R-project.org/package=MuMIn

[31] Lenth RV. Least-Squares Means: The R Package lsmeans. J Stat Softw. 2016;69: 1–33.

[32] Gaylor DW, Hopper FN. Estimating the Degrees of Freedom for Linear Combinations of Mean Squares by Satterthwaite’s Formula. Technometrics. 1969;11: 691–706. 10.1080/00401706.1969.10490732

[33] Westfall J, Kenny DA, Judd CM. Statistical power and optimal design in experiments in which samples of participants respond to samples of stimuli. J Exp Psychol Gen. 2014;143: 2020–2045. 10.1037/xge0000014 25111580

[34] Becchio C, Koul A, Ansuini C, Bertone C, Cavallo A. Seeing mental states: An experimental strategy for measuring the observability of other minds. Phys Life Rev. 2018;24: 67–80. 10.1016/j.plrev.2017.10.002 29066076

[35] Wassermann EM. Risk and safety of repetitive transcranial magnetic stimulation: report and suggested guidelines from the International Workshop on the Safety of Repetitive Transcranial Magnetic Stimulation, June 5–7, 1996. Electroencephalogr Clin Neurophysiol Potentials Sect. 1998;108: 1–16. 10.1016/S0168-5597(97)00096-8 9474057

[36] Rossi S, Hallett M, Rossini PM, Pascual-Leone A. Safety, ethical considerations, and application guidelines for the use of transcranial magnetic stimulation in clinical practice and research. Clin Neurophysiol. 2009;120: 2008–2039. 10.1016/j.clinph.2009.08.016 19833552 PMC3260536

[37] Brasil-Neto JP, Cohen LG, Panizza M, Nilsson J, Roth BJ, Hallett M. Optimal Focal Transcranial Magnetic Activation of the Human Motor Cortex: Effects of Coil Orientation, Shape of the Induced Current Pulse, and Stimulus Intensity. J Clin Neurophysiol. 1992;9: 132 1552001

[38] Mills KR, Boniface SJ, Schubert M. Magnetic brain stimulation with a double coil: the importance of coil orientation. Electroencephalogr Clin Neurophysiol Potentials Sect. 1992;85: 17–21. 10.1016/0168-5597(92)90096-T 1371739

[39] Betti S, Zani G, Guerra S, Castiello U, Sartori L. Reach-To-Grasp Movements: A Multimodal Techniques Study. Front Psychol. 2018;9 10.3389/fpsyg.2018.00009 29962993 PMC6013693

[40] Rossini PM, Barker AT, Berardelli A, Caramia MD, Caruso G, Cracco RQ, et al Non-invasive electrical and magnetic stimulation of the brain, spinal cord and roots: basic principles and procedures for routine clinical application. Report of an IFCN committee. Electroencephalogr Clin Neurophysiol. 1994;91: 79–92. 10.1016/0013-4694(94)90029-9 7519144

[41] Betti S, Castiello U, Guerra S, Sartori L. Overt orienting of spatial attention and corticospinal excitability during action observation are unrelated. PLOS ONE. 2017;12: e0173114 10.1371/journal.pone.0173114 28319191 PMC5358745

[42] D’Innocenzo G, Gonzalez CC, Nowicky AV, Williams AM, Bishop DT. Motor resonance during action observation is gaze-contingent: A TMS study. Neuropsychologia. 2017;103: 77–86. 10.1016/j.neuropsychologia.2017.07.017 28720525

[43] Wright DJ, Wood G, Franklin ZC, Marshall B, Riach M, Holmes PS. Directing visual attention during action observation modulates corticospinal excitability. PLOS ONE. 2018;13: e0190165 10.1371/journal.pone.0190165 29304044 PMC5755785

[44] Naish KR, Houston-Price C, Bremner AJ, Holmes NP. Effects of action observation on corticospinal excitability: Muscle specificity, direction, and timing of the mirror response. Neuropsychologia. 2014;64: 331–348. 10.1016/j.neuropsychologia.2014.09.034 25281883

[45] Sartori L, Becchio C, Bulgheroni M, Castiello U. Modulation of the action control system by social intention: unexpected social requests override preplanned action. J Exp Psychol Hum Percept Perform. 2009;35: 1490–1500. 10.1037/a0015777 19803652

[46] Salmelin R, Hámáaláinen M, Kajola M, Hari R. Functional Segregation of Movement-Related Rhythmic Activity in the Human Brain. NeuroImage. 1995;2: 237–243. 10.1006/nimg.1995.1031 9343608

[47] Jurkiewicz MT, Gaetz WC, Bostan AC, Cheyne D. Post-movement beta rebound is generated in motor cortex: Evidence from neuromagnetic recordings. NeuroImage. 2006;32: 1281–1289. 10.1016/j.neuroimage.2006.06.005 16863693

[48] Schuch S, Bayliss AP, Klein C, Tipper SP. Attention modulates motor system activation during action observation: evidence for inhibitory rebound. Exp Brain Res. 2010;205: 235–249. 10.1007/s00221-010-2358-4 20644919 PMC2914260

[49] Hummel F, Andres F, Altenmüller E, Dichgans J, Gerloff C. Inhibitory control of acquired motor programmes in the human brain. Brain. 2002;125: 404–420. 10.1093/brain/awf030 11844740

[50] Zarkowski P, Shin CJ, Dang T, Russo J, Avery D. EEG and the variance of motor evoked potential amplitude. Clin EEG Neurosci. 2006;37: 247–251. 10.1177/155005940603700316 16929713

[51] Sauseng P, Klimesch W, Gerloff C, Hummel FC. Spontaneous locally restricted EEG alpha activity determines cortical excitability in the motor cortex. Neuropsychologia. 2009;47: 284–288. 10.1016/j.neuropsychologia.2008.07.021 18722393

[52] Naish KR, Obhi SS. Self-selected conscious strategies do not modulate motor cortical output during action observation. J Neurophysiol. 2015;114: 2278–2284. 10.1152/jn.00518.2015 26311182 PMC4604217

[53] Baldissera F, Cavallari P, Craighero L, Fadiga L. Modulation of spinal excitability during observation of hand actions in humans. Eur J Neurosci. 2001;13: 190–194. 10.1046/j.0953-816x.2000.01368.x 11135017

[54] Mukamel R, Ekstrom AD, Kaplan J, Iacoboni M, Fried I. Single-Neuron Responses in Humans during Execution and Observation of Actions. Curr Biol. 2010;20: 750–756. 10.1016/j.cub.2010.02.045 20381353 PMC2904852

[55] Stamos AV, Savaki HE, Raos V. The Spinal Substrate of the Suppression of Action during Action Observation. J Neurosci. 2010;30: 11605–11611. 10.1523/JNEUROSCI.2067-10.2010 20810881 PMC6633417

[56] Vigneswaran G, Philipp R, Lemon RN, Kraskov A. M1 Corticospinal Mirror Neurons and Their Role in Movement Suppression during Action Observation. Curr Biol. 2013;23: 236–243. 10.1016/j.cub.2012.12.006 23290556 PMC3566480

[57] Hardwick RM, McAllister CJ, Holmes PS, Edwards MG. Transcranial magnetic stimulation reveals modulation of corticospinal excitability when observing actions with the intention to imitate: Intention to imitate modulates action observation. Eur J Neurosci. 2012;35: 1475–1480. 10.1111/j.1460-9568.2012.08046.x 22519854

[58] Tipper SP. From observation to action simulation: The role of attention, eye-gaze, emotion, and body state. Q J Exp Psychol. 2010;63: 2081–2105. 10.1080/17470211003624002 20721814 PMC2988435

[59] Bach P, Peatfield NA, Tipper SP. Focusing on body sites: the role of spatial attention in action perception. Exp Brain Res. 2007;178: 509–517. 10.1007/s00221-006-0756-4 17091293 PMC2079350

[60] Chong TT-J, Williams MA, Cunnington R, Mattingley JB. Selective attention modulates inferior frontal gyrus activity during action observation. NeuroImage. 2008;40: 298–307. 10.1016/j.neuroimage.2007.11.030 18178107

[61] Chong TT-J, Cunnington R, Williams MA, Mattingley JB. The role of selective attention in matching observed and executed actions. Neuropsychologia. 2009;47: 786–795. 10.1016/j.neuropsychologia.2008.12.008 19124033

[62] Sartori L, Cavallo A, Bucchioni G, Castiello U. Corticospinal excitability is specifically modulated by the social dimension of observed actions. Exp Brain Res. 2011;211: 557 10.1007/s00221-011-2650-y 21472443

[63] Flach R, Press C, Badets A, Heyes C. Shaking hands: Priming by social action effects. Br J Psychol. 2010;101: 739–749. 10.1348/000712609X484595 20100397

[64] Bunday KL, Lemon RN, Kilner JM, Davare M, Orban GA. Grasp-specific motor resonance is influenced by the visibility of the observed actor. Cortex. 2016;84: 43–54. 10.1016/j.cortex.2016.09.002 27697663 PMC5084682

[65] Cole GG, Skarratt PA, Kuhn G. Real Person Interaction in Visual Attention Research. Eur Psychol. 2016;21: 141–149. 10.1027/1016-9040/a000243

[66] Chemero A. An Outline of a Theory of Affordances. Ecol Psychol. 2003;15: 181–195. 10.1207/S15326969ECO1502_5

[67] Gibson JJ. The Ecological Approach to Visual Perception. Boston: Houghton Mifflin; 1979.

[68] Tipper SP, Paul MA, Hayes AE. Vision-for-action: The effects of object property discrimination and action state on affordance compatibility effects. Psychon Bull Rev. 2006;13: 493–498. 10.3758/BF03193875 17048736

[69] Borghi AM, Flumini A, Natraj N, Wheaton LA. One hand, two objects: Emergence of affordance in contexts. Brain Cogn. 2012;80: 64–73. 10.1016/j.bandc.2012.04.007 22634033

[70] Scorolli C, Miatton M, Wheaton LA, Borghi AM. I give you a cup, I get a cup: A kinematic study on social intention. Neuropsychologia. 2014;57: 196–204. 10.1016/j.neuropsychologia.2014.03.006 24680723

[71] Borghi AM. Affordances, context and sociality. Synthese. 2018; 10.1007/s11229-018-02044-1

[72] Dezecache G, Conty L, Grèzes J. Social affordances: Is the mirror neuron system involved? Behav Brain Sci. 2013;36: 417–418. 10.1017/S0140525X12001872 23883746

[73] Gallagher HL, Frith CD. Dissociable neural pathways for the perception and recognition of expressive and instrumental gestures. Neuropsychologia. 2004;42: 1725–1736. 10.1016/j.neuropsychologia.2004.05.006 15351623

[74] Sartori L. Complementary Actions In: Obhi SS, Cross ES, editors. Shared Representations. Cambridge: Cambridge University Press; 2016 pp. 392–416. 10.1017/CBO9781107279353.020

[75] Sartori L, Bucchioni G, Castiello U. When emulation becomes reciprocity. Soc Cogn Affect Neurosci. 2013;8: 662–669. 10.1093/scan/nss044 22490925 PMC3739911

[76] Sebanz N, Shiffrar M. Detecting deception in a bluffing body: The role of expertise. Psychon Bull Rev. 2009;16: 170–175. 10.3758/PBR.16.1.170 19145029

[77] Finisguerra A, Amoruso L, Makris S, Urgesi C. Dissociated Representations of Deceptive Intentions and Kinematic Adaptations in the Observer’s Motor System. Cereb Cortex. 2018;28: 33–47. 10.1093/cercor/bhw346 29253254

[78] Wokke ME, Knot SL, Fouad A, Richard Ridderinkhof K. Conflict in the kitchen: Contextual modulation of responsiveness to affordances. Conscious Cogn. 2016;40: 141–146. 10.1016/j.concog.2016.01.007 26821243

[79] Reader AT, Holmes NP. Examining ecological validity in social interaction: problems of visual fidelity, gaze, and social potential. Cult Brain. 2016;4: 134–146. 10.1007/s40167-016-0041-8 27867831 PMC5095160

[80] Kingstone A, Smilek D, Ristic J, Kelland Friesen C, Eastwood JD. Attention, Researchers! It Is Time to Take a Look at the Real World. Curr Dir Psychol Sci. 2003;12: 176–180. 10.1111/1467-8721.01255

