# Supplementary material for: Gaze and body cues interplay during interactive requests
Source: PLoS One. 2019 Oct 21;14(10):e0223591. doi: 10.1371/journal.pone.0223591 (PMC6802846; doi:10.1371/journal.pone.0223591)
Supplement: S1 Table — Means and standard deviations (in brackets) for each time epoch (T1, T2), Action (Interactive, Non Interactive), Gaze direction (Convergent, Divergent) and AOI (Head, Hand, Block, Mug) are presented. (DOCX) [file pone.0223591.s001.docx]

**Supplementary Table 1**: Descriptive statistics for the Fixation Duration variable for each time epoch (T1, T2), Action (Interactive request, Non Interactive), Gaze direction (Convergent, Divergent) and AOI (Head, Hand, Block, Mug). Means and standard deviations (in brackets) are presented.

|  |  | **AOI** | | | |
| --- | --- | --- | --- | --- | --- |
|  | **CONDITION** | **Head** | **Hand** | **Block** | **Mug** |
| **FIXATION DURATION**  **T1** | **Interactive Convergent Gaze** | 4.35 (1.84) | 2.15 (1.38) | 2.21 (1.86) | 0.10 (0.40) |
|  | **Interactive Divergent Gaze** | 4.12 (1.88) | 2.55 (1.25) | 1.89 (1.59) | 0.10 (0.37) |
|  | **Non Interactive Convergent Gaze** | 3.73(1.90) | 3.01 (1.23) | 2.25 (1.53) | 0.06 (0.26) |
|  | **Non Interactive Divergent Gaze** | 3.74 (1.49) | 2.40 (1.72) | 2.04 (1.86) | 0.04 (0.12) |
| **FIXATION DURATION**  **T2** | **Interactive Convergent Gaze** | 1.98 (1.41) | 1.39 (1.13) | 0.06 (0.15) | 0.26 (0.46) |
|  | **Interactive Divergent Gaze** | 2.20 (0.93) | 1.57 (0.98) | 0.29 (0.44) | 0.13 (0.24) |
|  | **Non Interactive Convergent Gaze** | 1.44 (0.99) | 0.89 (0.51) | 1.26 (0.98) | 0.06 (0.18) |
|  | **Non Interactive Divergent Gaze** | 2.60 (0.89) | 0.69 (0.68) | 0.39 (0.50) | 0.49 (0.66) |
